# TRK immunohistochemistry in pediatric rhabdomyosarcomas: diagnostic and prognostic utility and limitations

**DOI:** 10.3389/pore.2026.1612374

**Published:** 2026-07-17

**Authors:** Renáta Lajti, Katalin Dezső, Zoltán Sápi, Gergő Papp, Anna Mohás, Bence Bukovszky, Sándor Szabó, Luca Felkai, Monika Csóka

**Affiliations:** 1 Pediatric Center Tűzoltó Street Department, Faculty of Medicine, Semmelweis University, Budapest, Hungary; 2 Department of Pathology and Experimental Cancer Research, Faculty of Medicine, Semmelweis University, Budapest, Hungary

**Keywords:** immunohistochemistry, NTRK, rhabdomyosarcoma, soft tissue sarcoma, targeted therapy

## Abstract

Rhabdomyosarcoma is the third most common extracranial solid tumor in childhood. Treatment of patients is multimodal, based on systemic chemotherapy and local therapy by surgery and/or radiotherapy. Although standard therapies result in an overall survival of more than 75% for patients in the low-risk group, the prognosis for high-risk patients remains poor. For this reason, new therapeutic alternatives are needed. One such option is the use of specific Tropomyosin receptor kinase (TRK) inhibitors, which provides the opportunity of targeted therapeutic treatment for patients carrying *Neurotrophic Receptor Tyrosine Kinase* (*NTRK*) translocations. As the frequency of these aberrations is not yet known in rhabdomyosarcomas, the aim of this study was to map the pan-TRK expression profile of rhabdomyosarcomas by an affordable method and to investigate the link between the expression and the clinicopathological features. We studied samples of patients diagnosed with rhabdomyosarcoma at the Pediatric Center, Semmelweis University, Budapest, Hungary. Using immunohistochemistry, we found that pan-TRK expression was detected in 38% of the cases studied. The expression was present in a significantly higher proportion of samples in the unfavorable, alveolar histological subtype. However, no *NTRK* fusion was detected in the 17 TRK expressing samples.

## Introduction

Soft tissue sarcomas account for about 8%–10% of childhood tumors, the most common being rhabdomyosarcoma (RMS) [1]. It ranks behind neuroblastoma and Wilms tumor as the third most common extracranial solid tumor in children [2]. RMS is a malignant tumor of mesenchymal origin [2]. Patients typically present with symptoms due to the growth of the tumor and its impact on surrounding structures. The tumor can occur anywhere in the body, although the most common sites are the head and neck region, the urogenital tract and the extremities [3, 4]. According to the World Health Organisation [5] 2020 classification, there are four histological subtypes: embryonal RMS (RME), alveolar RMS (RMA), spindle-cell/sclerosing RMS and pleiomorphic RMS, which are determinants of prognosis [5]. Previously it has been shown that the worse outcome of alveolar RMS is associated with the presence of the paired box 3 [6]/forkhead box protein O1 (FOXO1) or the paired box 7 (PAX7)/FOXO1 fusion gene and PAX/FOXO fusion negative alveolar RMS behaves in a way similar to the favorable embryonal-type RMS. Fusion status is now proposed to be used in prognostic stratification rather than histology [7]. However, more recent studies have identified additional, less common fusion partners involving PAX genes, such as PAX3/nuclear receptor coactivator 1 (NCOA1), PAX3/nuclear receptor coactivator 2 (NCOA2) and PAX3/INO80 Complex Subunit D (INO80D) [6]. Furthermore, distinct non-PAX gene fusions have been described in spindle cell RMS, which are linked to different clinical features and generally better outcome. In Hungary, patients are treated according to the Cooperative Weichteilsarkom Studiengruppe (CWS) 2012 guidelines [8]. Risk stratification is based on pathology, localization, tumor size, lymph node involvement, presence of distant metastases, age and primary surgical findings [7]. Multimodal treatment consists of a combination of surgical treatment, chemotherapy, and radiotherapy [1]. Although the overall survival of low-risk patients is good, the 5-year progression-free survival of high-risk patients is only 45%–65%. Survival of patients with primary metastases is even poorer, at only 30%. The second-line treatment in case of relapse is still not defined. In some cases, surgical resection, intensification of chemotherapy or radiotherapy can be effective, but in many cases, this is insufficient to achieve remission and is a burden for patients because of serious side effects [9]. For these patients, the use of Tropomyosin receptor kinase (TRK) inhibitors may represent a new therapeutic approach.

In recent years, fusions of the *Neurotrophic Receptor Tyrosine Kinase* (*NTRK*) 1-3 genes have been detected in several tumors [10, 11]. The *NTRK* genes encode three TRK proteins (Tropomyosin receptor kinase A (TRKA), Tropomyosin receptor kinase B (TRKB) and Tropomyosin receptor kinase C (TRKC)), which are transmembrane receptors activated by a group of four growth factors called neurotrophins [12]. The ligand of TRKA is neuronal growth factor, that of TRKB is brain-derived neurotrophic factor, or neurotrophin 4, and the ligand of TRKC is neurotrophin 3 (12). Normally, activation of the kinase domain by ligands binding to their receptors triggers activation of downstream signaling pathways [10]. The main downstream effectors are the mitogen-activated protein kinase (MAPK) the phosphatidylinositol 3′-kinase [13] and the protein kinase C (PKC) pathways [10]. TRK proteins are physiologically expressed in neuronal tissues during embryonal development and in adults as well and play a role in the development and function of nerve cells [10].

The most common genetic variation affecting *NTRK* genes is inter- or intrachromosomal translocation [10]. To date, more than 80 possible gene partners have been described [14]. Translocation results in a fusion gene with a chimeric protein, which has a ligand-independent tyrosine kinase activity and constant activation of the signaling pathway [10]. This causes cell disruption leading to tumorigenesis. Another possible oncogenic mechanism is an activating mutation affecting *NTRK* genes [10]. In colorectal tumors and non-small cell lung cancers, inactivation of the TRKB protein, which is impaired by mutations in *NTRK*2, has been shown to result in reduced tumor formation [10]. In some cases, however, the products of mutant *NTRK* genes are not different from wild-type proteins, so the presence of these gene aberrations does not affect the risk of tumor formation [10]. Consequently, the possible role of *NTRK* mutations in inducing tumorigenesis is not yet fully understood. Another mechanism promoting tumorigenesis is alternative splicing. Oncogenic splice variants of the TRKA protein have been detected in human neuroblastoma samples [10]. The variant protein lacked the extracellular ligand-binding domain, resulting in its ability to spontaneously dimerize and become ligand-independent [10]. Finally, another possibility inducing tumorigenesis is the overexpression of TRK proteins. Increased tumor cell proliferation and migration has been shown in mammary, lung and skin cancer tissues to be due to the overexpression of TRK proteins and the consequent increased tyrosine kinase activity [10]. In neuroblastoma patients, protein expression is associated with tumor aggressiveness [10]. Increased TRKA and TRKC expression has been shown to be predictive of favorable outcome in this tumor type, whereas TRKB is characteristic of poorly differentiated tumors in most cases [10].

*NTRK* gene fusions are present in both pediatric and adult tumors, but research suggests that they may play a more significant role in the development of rare pediatric tumors [15]. They may occur at high frequency in certain rare childhood tumors (such as infantile fibrosarcoma) but may also occur at lower frequency in childhood malignancies with higher incidence (such as neuroblastoma or retinoblastoma) [12, 16, 17].

Although the first *NTRK* gene was discovered in 1982, the gene family has only become a focus of research in the last 5–6 years [18]. This is due to the emergence of targeted TRK inhibitor therapy in recent years. Small-molecule TRK inhibitors are tumor agnostic agents, i.e., the use of drugs is not recommended according to histological subtype of the tumor, but only according to the presence of an *NTRK* gene fusion [19]. Several TRK inhibitors have been approved for use worldwide, and many are currently in phase 1 or 2 trials [19]. These drugs are orally administered, well tolerated, have a minimal side effect profile, and act at very low concentrations. As TRK proteins are physiologically expressed in nerve cells, side effects mainly affect the nervous system. The most common on-target side effects are ataxia, dizziness, appetite increase and paresthesia [10]. Off-target side effects have also been described as an elevation of liver enzyme levels, constipation, diarrhea, fatigue and nausea, but these are mostly grade 1 or 2 in severity and reversible [10]. The two best known first-generation TRK inhibitors are Entrectinib and Larotrectinib [19]. Both agents inhibit all three TRK receptors, and Entrectinib also inhibits two additional tyrosine kinases, anaplastic lymphoma kinase (ALK) and ROS proto-oncogene 1, receptor tyrosine kinase (ROS1) [19, 20]. The agents are currently used in pediatric and adult solid tumors where no other satisfactory treatment is available, i.e., in progressive, metastatic, locally advanced, or inoperable patients [21, 22].

The TRK expression profile of RMS has not been characterized yet. However, based on the efficacy of available targeted therapies and *NTRK* mutations described in other childhood malignancies, it seemed worthwhile to test RMS samples for TRK expression. The aim of our study was to map the TRK expression profile of histological specimens from patients treated for RMS in the past 15 years at the Pediatric Center, Semmelweis University, Budapest, Hungary. We also aimed to investigate the association between TRK expression and risk factors as well as other clinicopathological features. Overall, the main goal of this study was to identify patients who may possibly benefit from TRK inhibitor treatment in the case of relapse or progression in a cost-efficient way.

Several methods are available to identify *NTRK* abnormalities, including immunohistochemistry (IHC), fluorescence *in situ* hybridization (FISH), reverse transcriptase–polymerase chain reaction, or next-generation sequencing (NGS) [23, 24]. Detecting the present TRK protein by immunohistochemistry (IHC) could be a cost-effective, possible pre-screening method. Confirmation of *NTRK* gene fusions by genetic testing is mandatory when TRK inhibitors are used [21, 25].

In tumors where a common genetic abnormality is known or genetic testing is routinely performed, the presence of *NTRK* fusions can also be detected by this genetic test [26]. However, as the prevalence of this abnormality in rhabdomyosarcomas is unknown, the proposed algorithms suggest that it could be more reasonable to perform IHC as a first screening method, due to its cost-effectiveness and wide availability [27]. Therefore, we chose IHC for first line analysis of TRK expression in our study. Immunohistochemical staining was performed using the Ventana pan-TRK (EPR17341) assay as primary antibody. This antibody reacts with the conserved, C-terminal peptide region of all three TRK proteins (TRKA, TRKB, TRKC), thus showing positivity for any present TRK protein [12]. With the use of this assay, a sensitivity of 97% and a specificity of 98% were described in previous studies [28]. However, it is important to note that these values were derived from studies across various tumor types and may not be directly applicable to sarcomas. A recent meta-analysis reported an overall false-negative rate of 18% for pan-TRK IHC, with NTRK3 fusions demonstrating the highest false-negative rate (27%) compared to NTRK1 (6%) and NTRK2 (14%) fusions [29] Moreover, both sensitivity and specificity of pan-TRK IHC have been shown to be particularly poor in sarcomas [30]. Therefore, a negative IHC result cannot reliably exclude an NTRK fusion, and this limitation should be considered when interpreting our findings. The high sensitivity is particularly important in this study due to the low prevalence of NTRK fusions, as patients with negative IHC results are not likely to be re-tested for this biomarker.

In addition to immunohistochemical analysis, IHC-positive cases underwent further molecular testing to confirm the presence of underlying NTRK gene fusions. Since pan-TRK immunohistochemistry may show positivity not only due to gene fusions but also as a result of wild-type TRK expression or other mechanisms, genetic confirmation is essential—especially in the context of considering TRK inhibitor therapy. Therefore, fluorescence *in situ* hybridization (FISH) and next-generation sequencing (NGS) were performed on the IHC-positive cases to verify the presence of clinically relevant NTRK rearrangements.

## Materials and methods

### Case selection

Our study was conducted on samples from children who had undergone treatment for RMS at the Pediatric Center, Semmelweis University, Budapest, Hungary. We enrolled all patients treated in this center between 2007 and 2022 with histological samples available, including those who were diagnosed elsewhere. This represents a total of 45 patients.

The sex distribution was almost equal among the patients included in this study, with 24 males and 21 females. Their age at diagnosis ranged from 0 to 17 years, with a median age of 4 years.

Of the rhabdomyosarcoma samples analyzed, 32 were embryonal, 8 alveolar and 5 belonged to other histological subtypes. Based on the risk classification of the treatment protocol, more than three quarters of the patients analyzed were in the high, very high risk or metastatic group [31]. Primary distant metastasis was present in 16 patients and recurrence was diagnosed in 9 cases. By the end of the study period, 9 patients had deceased. The clinicopathological features of the included cases are presented in Table 1.

**TABLE 1:** Clinicopathological characteristics of patients studied.

Clinicopathological features		No.	%
Sex	Female	21	46.7
	Male	24	53.3
Age	<10 years	33	73.3
	>10 years	12	26.7
Histological subtype	Embryonal RMS	32	71.1
	Alveolar RMS	8	17.8
	Spindle-cell/sclerosing RMS	5	11.1
	Pleiomorphic RMS	0	0
Tumor site	Favorable (orbit, genito-urinary non bladder/prostate, non-parameningeal head and neck tumors)	16	35.6
​	Unfavorable (orbit with bone arrosion and parameningeal tumors, genito-urinary bladder/prostate tumors or tumors at other sites not mentioned above)	29	64.4
Tumor size	<5 cm	16	35.6
	>5 cm	29	64.4
Nodal status	N0	25	55.6
	N1	20	44.4
Primary metastasis	Absent	29	64.4
	Present	16	35.6
Risk group	Low	0	0
	Standard	12	26.6
	High	16	35.6
	Very high	3	6.7
	Metastatic disease	14	31.1
Overall status	Complete remission	31	68.9
	Partial remission	0	0
	Stable disease	4	8.9
	Progressive disease	10	22.2

Abbreviations: RMS: rhabdomyosarcoma; (N0: no lymph node involvement; N1: lymph node involvement is present).

### Tissue collection

Sections were taken after collecting formalin-fixed paraffin-embedded (FFPE) histological blocks containing representative tumor tissue. In a subset of the samples, 16 in total (cases n1, n3, n4, n5, n6, n9, n11, n12, n13, n14, n15, n18, n19, n20, n21, and n25) tissue microarrays (TMAs) were prepared for the study. Individual sections were made for the remaining blocks.

### Hematoxylin-eosin staining

It should be noted that the primary antibody used in the IHC does not discriminate between wild-type and mutant TRK protein. Hematoxylin-eosin (HE) staining was performed on all sections to identify and localize tumor cells, ensuring that TRK expression was subsequently evaluated specifically in neoplastic cells on the corresponding immunostained sections.

### Detection of expression of TRK proteins by IHC

To detect the expression of TRK proteins in rhabdomyosarcoma cells, we performed IHC on the tumor samples. We prepared 4 μm thick sections from FFPE blocks. To evaluate TRK protein expression, we performed immunostaining using the Leica BOND-MAX Fully Automated IHC Staining System as recommended by the manufacturer. Epitope retrieval was performed with a pH 9 buffer at 100 °C for 20 min. The primary antibody (Ventana pan-TRK (EPR17341)) was added for a 120-min incubation. We used the Bond Polymer Refine Detection kit (Leica DS9800) for detection. The stained sections were digitized using a scanner (P1000-3DHistech) and analyzed for expression levels and cellular location of the protein using the CaseViewer software.

### Grading of immunohistochemical expression

Based on previous *NTRK* immunohistochemical studies, a sample is considered positive if at least 1% of the cells are stained [32]. This low threshold was chosen to maximize sensitivity in detecting potential fusion-positive cases. Positive samples were marked with 1, 2 or 3 crosses based on the percentage of tumor cells showing positive staining. Samples were scored + if staining was between 1% and 50%, ++ if staining was between 50%–75%, and +++ if staining was greater than 75% of cells.

### Validation

Following immunohistochemical analysis, further genetic testing was performed on the IHC positive samples. The intention was to clarify whether gene fusions could be detected behind the immunopositivity or whether the increased TRK expression was attributable to other causes.

Two distinct methods were used to assess the specificity of immunohistochemistry. Among the 17 IHC positive cases, fluorescence *in situ* hybridization analysis was performed on 14 samples. All three *NTRK* genes were tested using breakpoint assays to confirm the possible presence of translocations. FISH was performed on paraffin-embedded tissue sections using ZytoLight SPEC dual color breakpoint probes specific for the 3 *NTRK* genes (ZytoLight SPEC *NTRK*1 Dual Color Break Apart Probe (PL123), ZytoLight SPEC *NTRK*2 Dual Color Break Apart Probe (PL163), and ZytoLight SPEC *NTRK*3 Dual Color Break Apart Probe (PL164)). At least 100 cells per sample were checked for signal separation or its absence under fluorescence microscopy with excitation and emission filters.

In addition, next-generation sequencing (NGS) studies were performed for 5 patients. For these studies, patients were selected whose clinical condition and response to therapy warranted further exploration of alternative treatment options. In 2 of these cases, both FISH and NGS analyses were performed, as additional molecular testing was considered clinically justified despite negative FISH results. The Trusight Oncology 500TM (TSO500) assay was used to analyse the complete coding sequence of 523 genes. This panel includes both DNA and RNA sequencing components. The test panel includes genes most relevant to tumor pathogenesis and response to therapy. The study further evaluates copy number variations of therapeutic relevance for 59 additional genes and provides information on gene fusions and splice variants for 55 genes. Gene fusion detection was based on the RNA component of the assay. RNA was extracted from FFPE tissue and its quality was assessed prior to library preparation to ensure suitability for analysis.

### Statistical analysis

Due to the small number of cases, we used Fisher’s Exact test to assess the link between clinicopathological data and TRK expression. We set the null hypothesis of no significant link between clinicopathological features and immunohistochemical positivity. A significance level of 5% (p-value <0.05) was applied for all statistical tests.

## Results

### Pan-TRK IHC

Of the 45 samples tested, Pan-TRK expression was confirmed in 17 cases (38%). Table 2 shows the results distributed by the most important aspects of the CWS risk stratification.

**TABLE 2:** Results of pan-Trk staining by major risk factors and association of TRK positivity with risk factors and clinicopathological data.

Clinicopathological features		Trk positive cases (n = 17)		TRK negative cases (n = 28)		P-value
		No.	%	No.	%	
Sex	Female	10	59	11	39	0.230
	Male	7	41	17	61	
Age	<10 years	13	76	20	71	1.000
	>10 years	4	24	8	29	
**Histology**	**Favorable**	**10**	**59**	**27**	**96**	**0.003**
	**Unfavorable**	**7**	**41**	**1**	**4**	
Size	<5 cm	7	41	9	32	0.749
	>5 cm	10	59	19	68	
Site	Favourable	9	53	7	25	0.107
	Unfavourable	8	47	21	75	
Nodal status	N0	9	53	16	57	1.000
	N1	8	47	12	43	
Primary distant metasasis	Absent	12	71	17	61	0.541
	Present	5	29	11	39	
Risk group	Low risk	4	24	7	25	1.000
	High risk	13	76	21	75	
Recurrence	No	13	76	23	82	0.711
	Yes	4	24	5	18	
Survival	Yes	13	76	23	82	0.711
	No	4	24	5	18	

Abbreviations: RMS: rhabdomyosarcoma; N0: no lymph node involvement; N1: lymph node involvement is present. Bold values indicate statistically significant results.

The CWS 2012 guideline defines five risk groups: low risk, standard risk, high risk, very high risk, and metastatic disease. Based on the clinical course of the disease, patients in the low and standard risk groups generally have a significantly better prognosis compared to patients in the other categories. Therefore, for the purpose of simplifying statistical analyses, we classified these patients collectively as “low risk”, while patients in the high, very high, and metastatic groups were grouped as “high risk”. This simplified classification is used exclusively for statistical analysis and does not replace the full risk group categorization, which is detailed in Table 1.

We examined the relationship between the presence of pan-TRK expression and clinicopathological features. Fisher’s exact test was used to assess the relation between the two qualitative variables. Histological subtype showed a significant association with the presence of pan-TRK expression (p < 0.05). Samples from the unfavorable histology group showed a much higher proportion (88%) of TRK positivity compared to samples from the favorable histology group (27%), as indicated by the bolded values in Table 2. No other clinical features—including age, sex, tumor size, tumor site, nodal status, the presence of primary distant metastasis, risk group, recurrence, or survival—showed a statistically significant correlation with TRK expression status (p > 0.05). The association between the presence of TRK expression and clinicopathological features is shown in Table 2.

**FIGURE 1:**
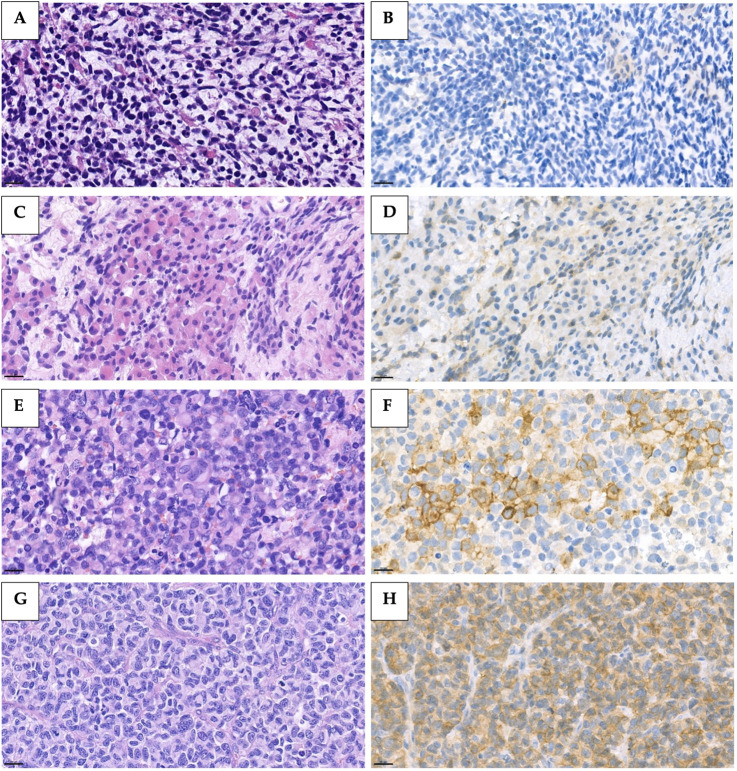
Different grades of pan-Trk immunohistochemical staining and corresponding HE stains, ×50 magnification. Picture **(A,B)** RMS patient n29 – negative IHC staining; **(C,D)** RMS patient n33 – weak IHC staining [less than 50% of cells stained (+)]; (**E,F)** RMS patient n18 – moderate IHC staining [50%–75% of cells stained (++)]; (**G,H)** RMS patient n40 – strong IHC staining [more than 75% of cells stained (+++)]. Images were acquired using CaseViewer 2.3 software (3DHistech Ltd., Budapest, Hungary). Scale bars show 20 μm.

Positive samples were graded according to the percentage of tumor cells showing positive staining. We found that a total of 6 samples (specifically cases n9, n14, n16, n25, n33, and n34) showed mild staining. In these cases, less than 50% of the tumor cells demonstrated positive staining, which we categorized as mild (+) staining. In addition, 4 samples (specifically cases n18, n36, n38, and n39) showed moderate staining, i.e. 50-75% of the cells were positively stained, and were therefore classified as moderate (++). Finally, 7 samples (specifically cases n7, n22, n35, n37, n40, n41, and n45) showed strong staining. As more than 75% of the tumor cells were positively stained in these cases, we categorized them as strong (+++) staining. Images of different grades of pan-Trk immunohistochemical staining and corresponding HE stains are shown in Figure 1.

**FIGURE 2:**
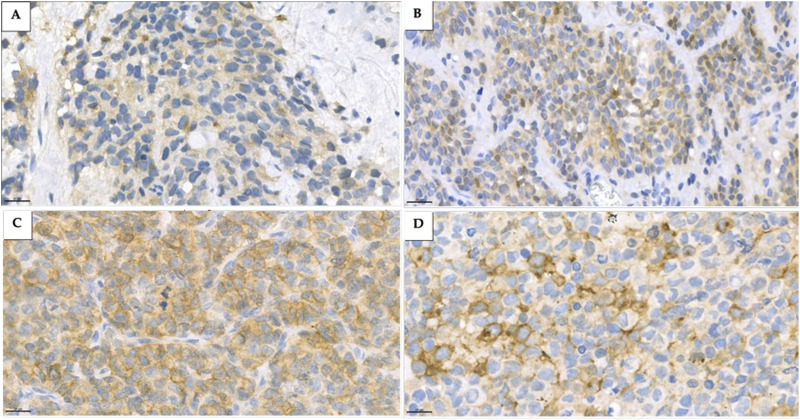
Cellular location of pan-Trk proteins, ×60 magnification. Image **(A)** of RMS patient n7 – cytoplasmatic staining; **(B)** RMS patient n22 **–** cytoplasmatic and nuclear staining; **(C)** RMS patient n40 – cytoplasmatic and membrane staining; **(D)** RMS patient n41 – cytoplasmatic, nuclear and membrane staining. Images were acquired with CaseViewer 2.3 software (3DHistech Ltd., Budapest, Hungary). Scale bars show 20 μm.

The TRK protein typically showed a cytoplasmic location in tumor cells (94%). In several cases, nuclear positivity (35%) and membrane positivity (35%) were also detected in addition to cytoplasmic staining. The significance of the cellular distribution of the TRK protein is not yet known. The immunohistochemical staining intensity of each positive sample and the cellular location of the expressed protein are shown in Table 3. Representative images illustrating the various subcellular localization patterns of pan-TRK immunostaining are presented in Figure 2.

**TABLE 3:** Staining intensity and cellular location of pan-Trk proteins in positive samples.

Pan-Trk-positive cases	Histology	Intensity of staining	Cellular location of Trk protein
n9	RME	+	Cytoplasmic
n14		+	Cytoplasmic + nuclear + membrane
n18		++	Cytoplasmic + membrane
n22		+++	Cytoplasmic + nuclear
n34		+	Cytoplasmic
n35		+++	Cytoplasmic
n36		++	Cytoplasmic
n38		++	Cytoplasmic
n39		++	Cytoplasmic
n40		+++	Cytoplasmic + membrane
n7	RMA	++++	Cytoplasmic
n16		+	Nuclear
n25		+	Cytoplasmic
n33		+	Cytoplasmic
n37		+++	Cytoplasmic + nuclear + membrane
n41		+++	Cytoplasmic + nuclear + membrane
n45		+++	Cytoplasmic + nuclear + membrane

We were also interested in whether the intracellular location of the TRK protein could have any clinical significance. Therefore, we investigated whether there was a significant association between the intracellular location of the TRK protein and clinicopathological features. To facilitate this analysis, the staining patterns were dichotomized into two groups: the ‘Cytoplasmic only’ group and the ‘Complex/Other’ group. This specific dichotomization was chosen because the ‘Cytoplasmic only’ pattern was the most frequent observation in our cohort. By grouping all other rarer, more complex staining patterns (involving nuclear or membrane components) into a single ‘Complex/Other’ category, we ensured a statistically meaningful comparison while isolating the clinical impact of the most common, purely cytoplasmic expression against cases with additional intracellular localization.

As indicated in Table 4, our analysis demonstrated a statistically significant association between the staining pattern and tumor recurrence (p = 0.035), with no recurrence cases observed in the “Cytoplasmic only” group, as indicated by the bolded values in the table. No other clinical parameters reached statistical significance. This means that in our cohort TRK protein was predominantly located solely in the cytoplasm in non-recurrent tumors. The correlation between the cellular location of Trk proteins and clinicopathological features is shown in Table 4.

**TABLE 4:** Relation of risk factors and clinicopathological data to the cellular location of Trk proteins.

Fisher’s exact test Relation of risk factors and clinicopathological data to the cellular location of Trk proteins						
Clinicopathological features		Cytoplasmic only (n = 9)		Complex/other cellular location (n = 8)		P-value
		No.	%	No.	%	
Sex	Female	5	56	4	50	1.000
	Male	4	44	4	50	
Age	<10 years	5	56	5	62	1.000
	>10 years	4	44	3	38	
Histology	Favorable	4	44	4	50	1.000
	Unfavorable	5	56	4	50	
Size	<5 cm	3	33	3	38	1.000
	>5 cm	6	67	5	62	
Site	Favourable	5	56	4	50	1.000
	Unfavourable	4	44	4	50	
Nodal status	N0	5	56	4	50	1.000
	N1	4	44	4	50	
Primary distant metasasis	Absent	7	78	5	62	0.605
	Present	2	22	3	38	
Risk group	Low risk	3	33	2	25	1.000
	High risk	6	67	6	75	
**Recurrence**	**No**	**9**	**100**	**4**	**50**	**0.035**
	**Yes**	**0**	**0**	**4**	**50**	
Survival	Yes	8	89	7	87	1.000
	No	1	11	1	13	

Bold values indicate statistically significant results.

### Genetic testing

**FIGURE 3:**
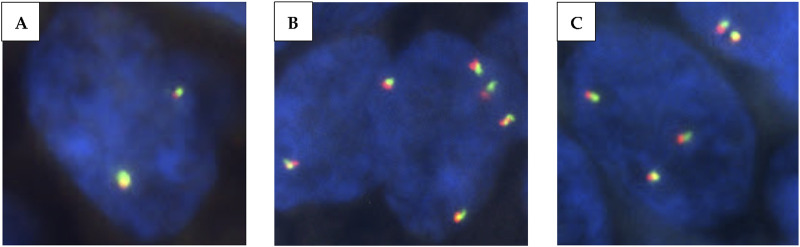
Fluorescence *in situ* hybridization images of *NTRK*1, 2, and 3 genes in a pan-Trk IHC positive tumor sample (case n45). Image **(A)** Non-rearranged NTRK1 signals using break-apart FISH probe; **(B)** Non-rearranged NTRK2 signals using break-apart FISH probe; **(C)** Non-rearranged NTRK3 signals using break-apart FISH probe. Original images.

Confirmatory genetic testing was performed in all 17 IHC positive cases, including 14 FISH tests and 5 NGS analyses. In 4 cases, however, evaluable FISH results could not be obtained, presumably due to preanalytical factors and compromised nucleic acid preservation associated with long-term storage of the archival FFPE tissue samples. Samples from 2 patients were tested by both methods. None of the successfully performed tests revealed *NTRK* gene fusions underlying the immunohistochemical positivity by either FISH or NGS. Furthermore, neither copy number variations nor pathogenic point mutations were identified in the sequences tested. The results of the confirmatory tests are shown in Table 5. Representative FISH images for *NTRK1, NTRK2*, and *NTRK3* genes in a pan-TRK IHC positive sample (case n45) are shown in Figure 3.

**TABLE 5:** Results of genetic testing on selected pan-TRK IHC positive samples.

Pan-Trk-positive cases	Histology	IHC		FISH	NGS		
		Intensity of staining	Cellular location of Trk protein	Gene fusions	Gene fusions	Copy number variations	Pathogenic point mutations
n7	RMA	+++	Cytoplasmic	Negative	-	-	-
n9	RME	+	Cytoplasmic	Unsuccessful	-	-	-
n14	RME	+	Cytoplasmic + nuclear + membrane	Unsuccessful	-	-	-
n16	RMA	+	Nuclear	Negative	-	-	-
n18	RME	++	Cytoplasmic + membrane	Unsuccessful	-	-	-
n22	RME	+++	Cytoplasmic + nuclear	-	Negative	Negative	Negative
n25	RMA	+	Cytoplasmic	Negative	-	-	-
n33	RMA	+	Cytoplasmic	Negative	Negative	Negative	Negative
n34	RME	+	Cytoplasmic	Unsuccessful	-	-	-
n35	RME	+++	Cytoplasmic	Negative	-	-	-
n36	RME	++	Cytoplasmic	Negative	-	-	-
n37	RMA	+++	Cytoplasmic + nuclear + membrane	Negative	-	-	-
n38	RME	++	Cytoplasmic	-	Negative	Negative	Negative
n39	RME	++	Cytoplasmic	Negative	-	-	-
n40	RME	+++	Cytoplasmic + membrane	Negative	-	-	-
n41	RMA	+++	Cytoplasmic + nuclear + membrane	-	Negative	Negative	Negative
n45	RMA	+++	Cytoplasmic + nuclear + membrane	Negative	Negative	Negative	Negative

Abbreviations: IHC: immunohistochemistry; FISH: fluorescence *in situ* hybridization; NGS: next-generation sequencing; TRK: tropomyosin receptor kinase; RMA: alveolar rhabdomyosarcoma; RME: embryonal rhabdomyosarcoma.

## Discussion

In recent years, *NTRK* gene fusions have been detected in several tumor types. Besides being very common in some rare tumors and considered as drivers of oncogenesis, research suggests that they also occur at lower frequencies in higher incidence malignancies [12, 15]. The discovery of TRK inhibitors that selectively inhibit tyrosine kinases opens up new possibilities for advanced tumors previously thought to be untreatable [19, 21]. This is the importance of proving *NTRK* gene fusions. Our goal was to analyze the TRK expression profile in pediatric RMS and assess its association with the clinicopathological features. Additionally, we provide an overview of the available FISH and NGS data.

In our study, we detected an immunohistochemical positivity of 38% among our patients. TRK protein expression was present in a high percentage of rhabdomyosarcoma samples with unfavorable alveolar histological subtype. Despite the lower number of cases with unfavorable histology in this study, the presence of TRK expression was confirmed in 7 out of the 8 cases (87.5%). It is well known that patients with unfavorable tumors, i.e., patients with alveolar histological subtype are at high risk and have a more aggressive and severe clinical course than patients with favorable histological subtypes. This means that these patients may be more likely to require alternative therapeutic modalities, such as targeted therapies, and thus the proven presence of TRK expression is of great importance in their case.

However, as previously shown in several studies of various types of sarcomas, pan-TRK immunohistochemical positivity is associated with *NTRK* fusions in only a minority of cases [15, 30, 33]. Notably, no prior research has specifically addressed pan-TRK immunohistochemical expression of pediatric rhabdomyosarcomas. Our confirmatory genetic tests have shown results in accordance with the existing literature on other sarcoma subtypes. Our findings suggest that in pediatric rhabdomyosarcomas the specificity of the pan-TRK immunohistochemical staining may be low. Among all tested IHC-positive cases, no evidence of NTRK gene fusion underlying the immunohistochemical positivity was found. In 5 cases, other genetic alterations, such as copy number variations and pathogenic point mutations were excluded as well. However, the small case number necessitates further investigations in the future. Nevertheless, as immunohistochemistry is a much more affordable and a more accessible method in comparison to genetic testing, its use as a screening method should continue to be considered.

Regarding the other clinicopathological features examined, no significant association was identified between these characteristics and immunohistochemical positivity. There was no statistically significant association of pan-TRK IHC positivity with sex, age, tumor size and tumor location, lymph node status, presence of primary distant metastasis, risk classification, relapse or survival.

In a significant proportion of our IHC positive samples, cytoplasmic staining was observed, accompanied by nuclear or membrane positivity. This is consistent with findings described in previous studies of pan-TRK immunohistochemistry of sarcomas [30]. Literature data suggest that the intracellular location of the fusion TRK protein depends on the fusion partner [34, 35]. However, as no genetically confirmed NTRK fusions were identified in our cohort, we cannot definitively attribute the observed subcellular staining patterns to specific fusion partners. To date, the significance of the intracellular location of TRK protein on tumor aggressiveness or therapeutic response is not fully understood. In our study, tumor recurrence showed a significant correlation with the cellular location of TRK protein. In our cohort, non-recurrent tumors exhibited exclusively cytoplasmic TRK localization. This association has not been described in previous studies, nor have similar results been found in the literature. Our results may be due to the small number of cases studied, and further studies with larger numbers of cases are recommended to confirm them. While this association cannot be linked to specific NTRK fusion partners without molecular confirmation, the observed association between subcellular TRK localization and recurrence suggest potential utility as a prognostic marker independent of fusion status. This is consistent with emerging evidence demonstrating that subcellular protein localization can serve as an independent prognostic indicator in various malignancies, including prostate and colorectal cancer [36, 37]. As no previous research results or literature data can support this association in rhabdomyosarcomas, further studies with larger cohorts are recommended to further substantiate it and explore the biological mechanisms underlying this association.

This study has several limitations that should be acknowledged. To date, there are no studies published focusing on TRK expression in pediatric rhabdomyosarcomas. However, a number of data is available on pan-TRK immunohistochemical studies of other types of sarcomas. Previous studies have shown that the sensitivity and specificity of the used pan-TRK antibody may be lower in sarcomas than in other pediatric mesenchymal tumors [15, 30, 33]. Although it should be emphasized that sarcomas are a diverse group of diseases, within which rhabdomyosarcoma, especially the childhood type, is a distinct entity. The results obtained in this study should therefore be evaluated in the light of these previous findings. Additionally, as this study was based on retrospective archival material, detailed information on pre-analytical parameters such as cold ischemic time, fixation duration, and tissue processing was not available for all cases. These variables are known to significantly influence IHC staining intensity and may represent a potential confounding factor in the interpretation of staining differences. This limitation should be acknowledged when evaluating our results.

On the basis of our results, it should be noted that pan-TRK IHC demonstrated a 0% positive predictive value for detecting NTRK fusions in our cohort, as none of the IHC-positive cases harbored a confirmed NTRK fusion. To our knowledge, this is the first study to systematically evaluate pan-TRK immunohistochemistry specifically in pediatric rhabdomyosarcomas, and therefore no prior literature data exist to guide expectations regarding positive predictive value in this tumor type. In case of positive immunohistochemical findings, confirmation of the result by genetic testing, such as fluorescence *in situ* hybridization or next-generation sequencing is mandatory. A positive immunohistochemical result is not sufficient for the indication of the targeted therapy for several reasons. Increased TRK expression may be due to various genetic abnormalities, such as point mutations or amplification, for which the efficacy of TRK inhibitors has not yet been clinically proven. It should also be emphasized that, according to the studies reported so far on sarcomas, *NTRK* gene fusions are rarely confirmed as the cause of immunohistochemical positivity in this tumor type [15, 30, 33]. However, if the genetic test confirms the presence of the *NTRK* fusion, the patient may be a potential candidate for TRK inhibitor treatment in case of relapse or progression. Furthermore, investigation of the genetic alterations underlying pan-TRK IHC positive RMS cases may present an intriguing area for future research.

## Brief case report

**FIGURE 4:**
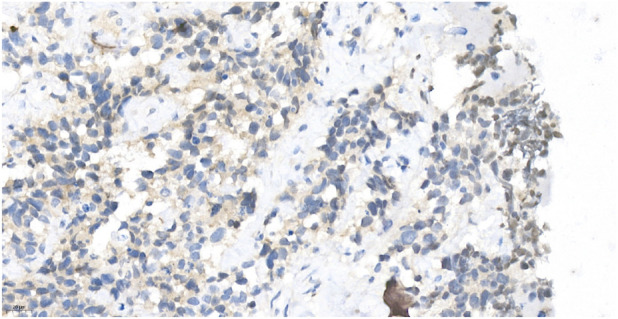
Pan-TRK immunohistochemistry of a pediatric rhabdomyosarcoma case (outside the study cohort) with confirmed NTRK fusion. The tumor cells demonstrate positive staining, subsequently verified by both FISH and NGS analysis. Original image. The image was acquired with CaseViewer 2.3 software (3DHistech Ltd., Budapest, Hungary). Scale bar shows 20 μm.

**FIGURE 5:**
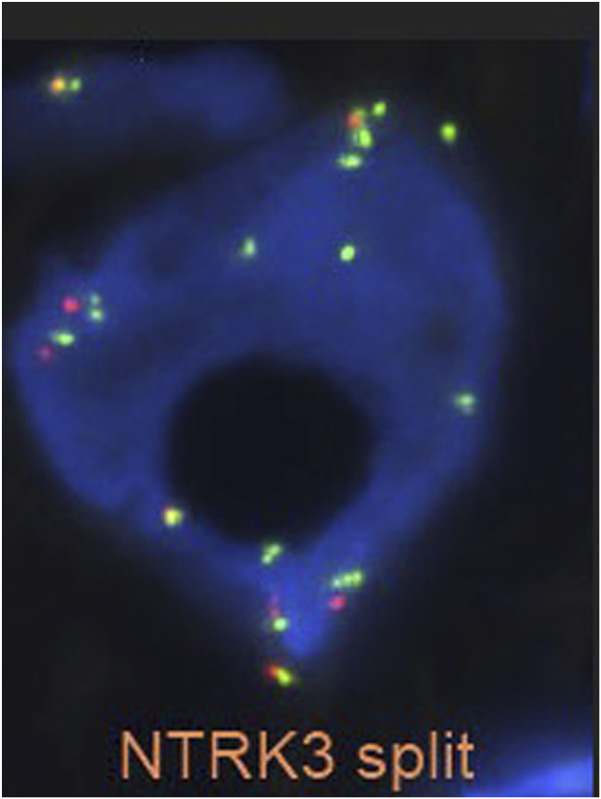
Rearranged *NTRK3* signals detected using break-apart FISH probe in the same case, confirming NTRK gene rearrangement. The presence of split signals indicates gene fusion, concordant with immunohistochemical and NGS findings. Original image.

Interestingly, during the course of our study—though outside the defined study period—we encountered a pediatric RMS case in our institution with TRK positivity confirmed by all three diagnostic modalities: IHC, FISH, and NGS. Initial immunohistochemical screening using a pan-TRK antibody revealed strong positive staining in the tumor cells (Figure 4). This finding was subsequently confirmed by fluorescence *in situ* hybridization (FISH), which detected rearranged NTRK3 signals using a break-apart probe, with the presence of split signals indicating gene fusion (Figure 5). Next-generation sequencing (NGS) further corroborated the NTRK gene rearrangement, providing definitive molecular confirmation. Based on this comprehensive, multi-modal diagnostic confirmation, the patient has been scheduled to receive TRK inhibitor therapy. Although this case fell outside the timeframe of our study cohort and was therefore not included in the statistical analysis, it serves as a compelling real-world example that underscores the clinical importance of integrating immunohistochemical screening with molecular confirmation techniques. Furthermore, it highlights the potential therapeutic implications of systematic TRK testing, particularly in high-risk pediatric patients who may benefit from targeted treatment options.

## Conclusion

The goal of our study was to map TRK protein expression in pediatric rhabdomyosarcomas using immunohistochemistry and analyze the link between immunohistochemical results and clinicopathological findings. We detected TRK positivity in 38% of cases, with a significantly higher frequency in the alveolar subtype. This histological variant, given its association with adverse prognosis, may represent a subgroup that could potentially benefit from targeted therapeutic strategies, such as TRK inhibitors.

Despite these encouraging findings, NTRK gene fusions could not be confirmed in any of the TRK-immunopositive cases using molecular testing. This discrepancy highlights the limitations of IHC as a surrogate marker for NTRK fusions in rhabdomyosarcomas. While IHC remains a widely accessible, cost-effective, and rapid initial screening method, our results suggest that it lacks sufficient specificity and cannot currently be recommended as a standalone diagnostic tool for selecting patients for TRK inhibitor therapy.

Genetic testing — such as NGS and/or FISH — remains the gold standard for the detection of NTRK gene fusions and for predicting the potential benefit of TRK-targeted treatments. To better define the diagnostic utility of IHC in this setting, larger-scale studies are required, incorporating systematic comparisons across IHC, FISH and NGS results. Such studies would help to clarify whether IHC can serve as a reliable pre-screening tool and under what conditions it may be integrated into routine diagnostic workflows.

In conclusion, while TRK protein expression does not appear to be an effective pre-screening tool for NTRK fusions in pediatric rhabdomyosarcomas based on our data, the observed associations between subcellular TRK localization and clinical parameters suggest potential alternative utility as a prognostic marker, independent of fusion status. Molecular confirmation remains essential for therapeutic decision-making. Further multicenter investigations with expanded sample sizes are warranted to validate our observations, to clarify the prognostic significance of subcellular TRK localization, and to optimize the diagnostic algorithm for TRK-targeted therapy in pediatric rhabdomyosarcoma.

## Data Availability

The raw data supporting the conclusions of this article will be made available by the authors, without undue reservation.

## Glossary

ALK: anaplastic lymphoma kinase
RMA: alveolar rhabdomyosarcoma
CWS: Cooperative Weichteilsarkom Study Group
RME: embryonal rhabdomyosarcoma
FFPE: formalin-fixed paraffin-embedded
FISH: fluorescence *in situ* hybridization
FOXO1: forkhead box protein O1
HE: hematoxylin-eosin
IHC: immunohistochemistry
INO80D: INO80 Complex Subunit D
MAPK: mitogen-activated protein kinase
N0: no lymph node involvement
N1: lymph node involvement is present
NCOA1: nuclear receptor coactivator 1
NCOA2: nuclear receptor coactivator 2
NGS: next-generation sequencing
NTRK: Neurotrophic Receptor Tyrosine Kinase
PAX3: paired box 3 gene
PAX7: paired box 7 gene
PI3K: phosphatidylinositol 3′-kinase
PKC: protein kinase C
RMA: alveolar rhabdomyosarcoma
RME: embryonal rhabdomyosarcoma
RMS: rhabdomyosarcoma
ROS1: ROS proto-oncogene 1, receptor tyrosine
TMA: tissue microarray
TRK: Tropomyosin receptor kinase
TRKA: Tropomyosin receptor kinase A
TRKB: Tropomyosin receptor kinase B
TRKC: Tropomyosin receptor kinase C
WHO: World Health Organization
